# Molecular epidemiology and multi-scale drivers of piscine myocarditis virus dispersal in salmon aquaculture

**DOI:** 10.1093/ve/veag020

**Published:** 2026-03-28

**Authors:** Mingli Zhao, Hélène Duault, Guillaume Fournié, Mari Aas Solheim, Svein Alexandersen, Chris Matthews, Silvia Soares, Abdullah Madhun, Marius Karlsen, Sarah C Hill

**Affiliations:** Department of Pathobiology and Population Sciences, Royal Veterinary College, University of London, Hatfield, Hertfordshire AL9 7TA, United Kingdom; Université de Lyon, INRAE, VetAgro Sup, UMR EPIA, Marcy l’Etoile 69280, France; Université de Lyon, INRAE, VetAgro Sup, UMR EPIA, Marcy l’Etoile 69280, France; Université Clermont Auvergne, INRAE, VetAgro Sup, UMR EPIA, Saint Genes Champanelle 63122, France; PHARMAQ AS, Harbitzalléen 2A, 0275 Oslo, Norway; PHARMAQ AS, Harbitzalléen 2A, 0275 Oslo, Norway; PHARMAQ Analytiq Limited, 22 Carsegate Rd, IV3 8EX Inverness, United Kingdom; Marine Laboratory, 375 Victoria Rd, Torry, Aberdeen, AB11 9DB Scotland, United Kingdom; Institute of Marine Research, Nordnesgaten 50, Nordnes, 5817 Bergen, Norway; PHARMAQ AS, Harbitzalléen 2A, 0275 Oslo, Norway; Department of Pathobiology and Population Sciences, Royal Veterinary College, University of London, Hatfield, Hertfordshire AL9 7TA, United Kingdom

**Keywords:** farmed salmon, cardiomyopathy syndrome; piscine myocarditis virus, phylodynamic analysis, evolutionary dynamics, virus dispersal, boat connectivity

## Abstract

Aquaculture is the fastest-growing animal food–producing sector for human consumption globally. Increasing population size, density, and interconnectivity among farms are expected to markedly increase disease outbreak risks, threatening sector sustainability. Infectious diseases cause around a third of observed mortality in farmed salmon in Norway. Cardiomyopathy syndrome (CMS), caused by piscine myocarditis virus (PMCV), is one of the most frequently detected diseases and often causes high mortality in the late phase of salmon production, compromising fish welfare and causing significant economic losses. Despite its impact, the limited understanding of PMCV dispersal and its drivers hampers effective risk mitigation interventions. To address this, we generated one of the largest genomic datasets for any aquatic viral pathogen, focusing on PMCV collected from Scotland (United Kingdom) and Norway. Combined with detailed metadata, this enabled one of the most comprehensive phylodynamic analyses of a fish virus. Phylodynamic analyses reveal that PMCV likely emerged in farmed salmon concurrent with the global expansion of aquaculture, with a most recent common ancestor around the 1970s–1990s. We identify a distinctive national clustering of virus genomes, reflecting intranational spread interspersed with multiple introductions of distinct PMCV lineages into Scotland. We assess factors influencing virus spread, highlighting ﻿the importance of geographic proximity and at-sea farm density, and ﻿the possible role of boat connectivity in facilitating long-distance dispersal events. Overall, our study emphasizes the importance of viral genomics in understanding the evolutionary and dispersal dynamics of aquatic pathogens and informs targeted biosecurity strategies to mitigate the growing risk of viral disease in expanding aquaculture systems.

## 1. Introduction

Aquaculture provides a significant share of the global animal protein supply and is the world’s fastest-growing animal-source food sector (Ottinger et al. 2016). Atlantic salmon (*Salmo salar* L.) is one of the most commercially important and widely farmed species (Boyd et al. 2022). Global production of farmed Atlantic salmon has exceeded 2.8 million tonnes in recent years, with Norway, Chile, and the UK among the leading producers (FAO 2025). However, the industry faces persistent challenges, with high mortality rates being one of the most significant. In Norway, approximately one-sixth of farmed salmon die before harvest, and infectious diseases account for around one-third of these losses (Moldal et al. 2025). Viral pathogens are a key cause of salmon mortality, compromising fish welfare and imposing substantial economic and sustainability burdens (Vedeler 2017, Valero and Cuesta 2023, Tapia et al. 2025).

Cardiomyopathy syndrome (CMS) is one of the most consequential viral diseases affecting Atlantic salmon (Ferguson et al. 1990, Moldal et al. 2025). CMS primarily kills large fish close to harvest, which leads to significant economic and sustainability losses, as substantial resources have already been invested in the care and feeding of these fish (Ferguson et al. 1990, Brun et al. 2003, Garseth et al. 2018, Svendsen et al. 2019, Moldal et al. 2025). In Norway, estimated direct annual losses attributed to CMS increased from approximately USD 4–7.85 million around 2000 to USD 80–105 million by 2007 (Brun et al. 2003, Vedeler 2017). The aetiological agent of CMS is piscine myocarditis virus (PMCV), a double-stranded RNA (dsRNA) virus belonging to the *Pistolviridae* family in the *Ghabrivirales* order (Haugland et al. 2011, Simmonds et al. 2024). Whereas most members of *Ghabrivirales* infect invertebrates, fungi, or protozoa, *Pistolviridae* are notable for infecting fish hosts (Mor and Phelps 2016, Sandlund et al. 2021, Simmonds et al. 2024). PMCV can be transmitted *via* extracellular release, as demonstrated in both *in vitro* and *in vivo* studies in Atlantic salmon (Haugland et al. 2011). To date, there is no conclusive evidence for nonfish hosts of PMCV (Nyman et al. 2024). The PMCV genome is 6688 bp and encodes three open reading frames (ORFs): ORF1 (capsid), ORF2 (RNA-dependent RNA polymerase; RdRp), and ORF3, which shares limited homology with the chemokine superfamily (Haugland et al. 2011, Sandlund et al. 2021).

CMS was first identified in Norway in 1985 (Amin and Trasti 1988) and has since been reported widely in Northern Europe including the Faroe Islands in 1992 (Poppe and Sande 1994, Poppe and Seierstad 2003), Ireland in 2012 (Rodger et al. 2014), and Scotland (UK) in 2000 (Rodger and Turnbull 2000). Preventing PMCV spread effectively requires a clearer understanding of its transmission routes, whether it spreads vertically (i.e. only from parent to offspring) or horizontally (i.e. between hosts regardless of descent), and the key drivers that facilitate its spread. Experimental evidence supports horizontal transmission, with PMCV spreading from infected to cohabitating Atlantic salmon (Haugland et al. 2011). In contrast, the contribution of vertical transmission *via* infected or contaminated gametes remains unresolved, with studies reporting divergent findings that may reflect differences in sampling and disinfection protocols (Wiik-Nielsen et al. 2012, Bang Jensen et al. 2019, Mikalsen et al. 2020).

Epidemiological studies have linked CMS risk to larger cohort size, higher infection pressure, and longer seawater production periods (Bang Jensen et al. 2013). However, several widely proposed drivers of PMCV spread remain poorly evaluated, including geographic proximity among farms, farm density, and network connectivity mediated by wellboats transporting fish among sites. Geographic proximity may increase the likelihood of local spread *via* water currents and other short-distance spread pathways, while farm density may elevate infection pressure by increasing the number of potential sources within a region and the frequency of indirect contacts. Wellboat-mediated network connectivity may further facilitate spread between distant sites by linking farms through repeated fish movements and associated operational contacts.

Despite the substantial impact of CMS, the evolutionary history and spatial spread of PMCV remain poorly resolved, primarily because genomic resources have been limited. Existing studies have typically relied on partial genomic regions (e.g. ORF1 and ORF3) or a small number of complete genomes and have focused largely on Norway, Ireland, and the Faroe Islands (Wiik-Nielsen et al. 2013, Tighe et al. 2019, Amono et al. 2024, Dahl et al. 2025). This has constrained time-scaled phylogenetic and phylodynamic inference, limiting robust reconstruction of lineage dispersal across space and time. Moreover, despite CMS being a leading cause of mortality in Scottish farmed salmon, and Scotland ranking among the top three salmon-producing countries globally, PMCV genome sequences from Scotland have not been available (Marine Directorate 2023, 2025), hindering inference on the timing, sources, and spatial patterns of PMCV introduction and onward spread within Scottish aquaculture.

Here, we address these gaps by generating the largest genome dataset for PMCV to date (311 near-complete genomes) from salmon aquaculture in Norway and Scotland. We developed an efficient amplicon-based whole-genome sequencing approach and integrated genomic data with detailed metadata to enable molecular clock-based phylodynamic and phylogeographic inference. This approach allows reconstruction of PMCV evolutionary history, characterization of multi-scale dispersal patterns, and evaluation of putative drivers of spread. Our findings inform improved surveillance and targeted biosecurity interventions for CMS. More broadly, our approach offers a template for applying genomic epidemiology to other important pathogens in aquaculture systems.

## 2. Materials and methods

### 2.1. Study ethics

All samples used in this study were residual diagnostic materials submitted to diagnostic or governmental laboratories for routine viral surveillance or in response to suspected or confirmed disease events. CMS diagnostic outcomes were not available for these samples. Residual samples and associated data from commercial diagnostic laboratories in Scotland and Norway were used with the written, informed consent of all producers. Residual samples from governmental laboratories in Scotland were used without the written, informed consent of producers; however, in these cases producer- and site-level identifiable data were not used. This study was approved by the appropriate Ethical Review Board at the Royal Veterinary College (URN: 2022 2098-3).

### 2.2. Samples and metadata

We processed a total of 235 samples from Norway and 111 samples from Scotland for sequencing. The Norwegian samples consisted of heart tissues collected from 81 distinct farm sites operated by 40 different companies across 10 production areas (designated as PA2–PA12; Fig. S1; Ådlandsvik 2015, Norwegian Ministry of Trade, Industry and Fisheries 2017), spanning the years 2012–23. The Scottish samples were collected from 36 fish farms between 2016 and 2024 from key production areas, including Orkney, the Shetland Islands, the Western Isles, and the west coast of mainland Scotland (Munro 2023). The number of samples per farm varied between 1 and 11, while the number of samples per cage ranged from 1 to 5. RNA extraction and reverse transcription quantitative polymerase chain reaction (RT-qPCR) assays were performed to detect PMCV RNA from heart tissues or a combination of heart and kidney tissues by the Marine Directorate, Pharmaq Analytiq (Scotland), or Pharmaq Analytiq AS (Norway). Details of the RNA extraction protocols and RT-qPCR assays were not provided by the diagnostic agencies, and are not reported here. Cycle threshold (Ct) values from RT-qPCR in the chosen samples ranged from 10.7 to 35.0, indicating varying levels of viral RNA in the specimens. Almost all but seven samples were collected from adult farmed salmon during the seawater phase of production; the remaining seven samples originated from freshwater broodstock. Anonymized metadata for successfully sequenced samples (genome coverage > 75%) are provided in Table S1.

### 2.3. Whole genome sequencing

We designed a set of 15 primer pairs to cover 6590 bp of the complete 6688 bp PMCV genome using the Primal Scheme tool (http://primal.zibraproject.org) and the published reference genome of PMCV (Table S2) (Haugland et al. 2011, Quick et al. 2017). Each primer pair targeted a polymerase chain reaction (PCR) amplicon of ~550 bp (544–573 bp) with roughly 100 bp overlap between adjacent amplicons.

We attempted whole-genome sequencing on 346 samples. Briefly (detailed protocols are available at https://www.protocols.io/edit/pcr-ngs-for-rna-virus-pmcv-dyk37uyn), cDNA was synthesized from total RNA using the LunaScript® RT SuperMix Kit (New England Biolabs, UK). Next, PCR amplification of corresponding double-stranded DNA (dsDNA) amplicons was conducted in two separate multiplex PCR reactions of nonoverlapping amplicons (Pool A and Pool B). PCR products were purified with 1× SPRIselect beads (Beckman Coulter, USA) and DNA concentrations quantified using a Qubit 3.0 fluorometer (Thermo Fisher Scientific, USA). We normalized pooled PCR products from Pool A and B to 5 ng/μl and prepared them for ligation using the NEBNext® Ultra™ II End Repair/dA-Tailing Module (New England Biolabs, UK). Subsequently, we ligated these products with native barcodes from the Native Barcoding Kit 96 (Oxford Nanopore Technologies (ONT), UK) using Blunt/TA Ligase Master Mix (NEB). Barcoded products were combined into a single library and purified with 0.4× SPRIselect beads (Beckman Coulter, USA). ONT native adapters were ligated to this library using the NEBNext® Quick Ligation Module, and a final 1 × AMPure beads (Beckman Coulter, USA) purification was performed. Libraries were quantified using a Qubit, and 20 ng from each library was loaded onto a MinION Flow Cell R9.4.1 or R10.4.1 for sequencing on an ONT MinION Mk1B.

### 2.4. Sequence assembly and alignment

We assembled sequences and generated consensus sequences using the ARTIC bioinformatics pipeline (https://github.com/artic-network/artic-ncov2019) with appropriately modified primer schemes. We performed basecalling with Guppy 5.0.11 with super-high accuracy settings, and demultiplexed reads with Guppy Barcoder 5.0.11, requiring barcodes on both ends of the reads (https://nanoporetech.com/document/Guppy-protocol). We filtered reads to retain only those with lengths between 400 and 700 bp. Primer sequences were removed, and the remaining reads were mapped to the reference genome of PMCV. Variants were called to create consensus sequences using Medaka v1.11.3 (https://github.com/nanoporetech/medaka). Sequences with genome coverage exceeding 75% were retained for further analysis.

We translated nucleotide sequences into amino acid sequences for each ORF. Multiple alignments of whole genome nucleotide sequences and amino acid sequences encoding ORFs were performed using MUSCLE 5.1 with default settings, as implemented in Geneious Prime (version 2024.0.5).

To validate the accuracy of viral genomic sequences obtained through the nanopore sequencing approach, we amplified a 545 bp region (nucleotide positions 2684–3228) from nine PMCV samples with primers: PMCV_7_LEFT (5′-ACAATGTGGCCACCATGAGG-3′) and PMCV_7_RIGHT (5′-CTGAATGCTCCCATCCATCCAG-3′). Reactions were run on a thermocycler with a heated lid set to 105°C under the following conditions: initial denaturation at 98°C for 1 min, followed by 30 cycles of 98°C for 30 s and 65°C for 5 min (combined annealing and extension), and a final hold at 4°C. PCR products were then sequenced by Sanger sequencing (GENEWIZ, UK). A 460 bp segment of high-quality sequence (positions 2719–3178) was obtained and used for comparison with the corresponding consensus sequences generated *via* nanopore sequencing.

### 2.5. Selection

To evaluate selection pressure, we assessed the ratio of nonsynonymous to synonymous substitutions (dN/dS) using multiple methods implemented within Datamonkey 2.0 (Weaver et al. 2018): Fixed Effects Likelihood (FEL) (Kosakovsky Pond and Frost 2005), Fast Unconstrained Bayesian Approximation (FUBAR) (Murrell et al. 2013), and Mixed Effects Model of Evolution (MEME) (Murrell et al. 2012). FUBAR and FEL both assume that the selection pressure for each site is constant along the phylogeny, while MEME aims to detect sites evolving under positive selection with a proportion of branches. These analyses were applied separately to the nucleotide alignments of each of the three ORFs. Sites with a dN/dS ratio > 1 and a *P*-value < .05 (for FEL), or a posterior probability >95% (for FUBAR), were considered to be under positive selection. Conversely, sites with a dN/dS ratio < 1 and a *P*-value < .05 (for FEL), or a posterior probability > 95% (for FUBAR), were considered to be under negative selection. Sites identified by both models were deemed to have undergone either pervasive positive or negative selection. In the MEME test, sites with a dN/dS ratio > 1 and a *P*-value < .05 were considered to be under episodic positive selection.

### 2.6. Recombination and clock-like signal

We used two different methods to check the absence of historical recombination between viruses, which could complicate whole genome phylogenetic analyses: RDP4 (Martin et al. 2015) and GARD (Genetic Algorithm for Recombination Detection) (Kosakovsky Pond et al. 2006), available in the Datamonkey 2.0 (Weaver et al. 2018).

To investigate the presence of an appropriate temporal signal and ‘clock-like’ evolution of the PMCV virus dataset, we performed a regression analysis of the root-to-tip genetic distance against the date of sampling in TempEst v1.5.3 (Rambaut et al. 2016). This analysis used a maximum-likelihood tree constructed with PMCV genomes from this study without a molecular clock assumption. We constructed this maximum likelihood (ML) phylogenetic tree using PhyML 3.0 (Guindon et al. 2010) and evaluated branch support through bootstrap analysis with 1000 pseudoreplicates (Felsenstein 1985). To determine the best-fit model of evolution, we used jModelTest 2.1 and selected a general time reversible model of nucleotide substitution rates with an estimated proportion of invariant sites and gamma-distributed rate variation for each nucleotide site (GTR + I + G4) model based on both the Akaike Information Criterion (AIC) and Bayesian Information Criterion (BIC) (Darriba et al. 2012). We also constructed an ML tree that included all PMCV genomes from this study and PMCV genome sequences with known sampling years available in NCBI GenBank (accessed on 1 May 2026). This analysis incorporated 43 recently published PMCV genome sequences from the Faroe Islands and 40 PMCV genomes from Norway. Additionally, a third ML tree was constructed to incorporate concatenated partial sequences (1478–2235 bp and 5668–6262 bp) of 32 PMCV genomes from Ireland (accessed on 1 May 2026; see Table S3 for NCBI accession numbers), for which all whole genomes were trimmed to the partial sequence length.

We then estimated a time-scaled phylogenetic tree with PMCV genomes from this study using the BEAST v1.10.4 (Drummond and Rambaut 2007, Suchard et al. 2018). Initially, we compared the appropriateness of two types of molecular clocks: the uncorrelated lognormal relaxed clock and the strict clock (Drummond et al. 2006, Ferreira and Suchard 2008), each alongside two coalescent models: constant population size (Kingman 1982, Griffiths and Tavare 1994) and Bayesian skygrid (Gill et al. 2013). All models employed a GTR + G4 substitution model (Shapiro et al. 2006). We ran at least two independent Markov Chain Monte Carlo (MCMC) analyses for 250 million steps, sampling every 25 000 steps. Path-sampling (Lartillot and Philippe 2006) and stepping-stone-sampling analyses (Fan et al. 2011, Xie et al. 2011) showed that the optimal model incorporated an uncorrelated relaxed clock (Drummond et al. 2006) with a constant size coalescent prior (Kingman 1982, Griffiths and Tavare 1994). To obtain posterior tree distributions, we ran two parallel MCMC chains, each consisting of 250 million steps with sampling every 25 000 steps. The convergence of the MCMC runs was evaluated by visual inspection of trace plots and effective sample sizes (ESSs) in Tracer v1.7.2 (Rambaut et al. 2018) (http://tree.bio.ed.ac.uk/software/tracer/). We then generated the maximum clade credibility (MCC) tree with TreeAnnotator v1.10.4 (Drummond and Rambaut 2007), discarding the initial 10% of samples as burn-in. Phylogenetic trees were visualized using the R package ggtree v3.15.0 (Yu et al. 2017). We also estimated the evolutionary rates of the three ORFs using two parallel MCMC runs with unlinked clock models, an uncorrelated relaxed clock, and a constant population size coalescent prior in the BEAST v1.10.4.

Additionally, we constructed a time-scaled phylogenetic tree that included all PMCV genomes from this study and 43 recently published PMCV genome sequences from the Faroe Islands and 40 PMCV genomes from Norway available in NCBI GenBank (see Table S3 for NCBI accession numbers).

### 2.7. Tip-association tests

We used the Bayesian Tip-association Significance Testing (BaTS) program v1.0 (Parker et al. 2008) to examine the clustering of traits among the tips of the PMCV phylogenetic tree. We initially assessed whether sequences clustered together based on the country of sampling (Norway or Scotland). Subsequently, we investigated whether sequences clustered by production areas within each country; these analyses were performed separately for Norway and Scotland due to significant clustering by country observed in the initial analysis. To test the clustering of traits among production areas, we analysed both the entire set of PMCV genomes and a subset containing only one genome per sampled farm site, ensuring the avoidance of farm-based clustering bias. Finally, we evaluated whether sequences clustered by the broodstock producer that supplied the salmon eggs for each fish cohort. For this test, we again randomly selected one genome with a known egg producer from each sampled farm site in Norway to prevent farm-based clustering. The significance of trait clustering was determined by comparing the observed association index (AI) and parsimony score (PS) from 1000 posterior samples of the PMCV BEAST tree against null distributions generated from 1000 randomizations of traits among the tips of each sampled PMCV tree.

### 2.8. Continuous phylogeographic analysis

We estimated the dispersal history of PMCV lineages using the continuous phylogeographic method implemented in BEAST v1.10.4 (Lemey et al. 2010, Suchard et al. 2018). For this analysis, only samples with known latitude and longitude coordinates were included, consisting of 220 Norwegian samples and 51 Scottish samples. For the inference of spatial locations at ancestral nodes, we applied a Cauchy relaxed random walk model. To obtain posterior tree distributions, two parallel MCMC chains were run for 250 million steps, with sampling every 25 000 steps. The maximum clade credibility tree was annotated using TreeAnnotator, discarding the initial 10% of samples as burn-in. To visualize the spatiotemporal information contained in the phylogenetic trees inferred with BEAST, we used the R package seraphim 1.0 (Dellicour et al. 2016). Data from 1000 trees sampled from the postburn-in posterior distribution were extracted. Seraphim was then employed to calculate dispersal statistics, including mean branch dispersal velocity, weighted branch dispersal velocity, mean diffusion coefficient, and weighted diffusion coefficient, based on the phylogenetic branches. Dispersal velocity refers to the rate at which viral lineages move through space over time, quantifying the linear distance covered by these lineages per unit of time across their phylogenetic history. The diffusion coefficient measures the rate at which viral lineages expand their geographic range, or the ‘diffusivity’, reflecting how quickly they invade a 2D space (Dellicour et al. 2024).

### 2.9. Spatial predictors of piscine myocarditis virus dispersal in Norway

To investigate which covariates are associated with the movement of PMCV viral lineages, we employed a generalized linear model (GLM) extension of a phylogeographic discrete trait analysis (DTA) in BEAST v1.10.4 (Drummond and Rambaut 2007, Faria et al. 2013, Suchard et al. 2018). Each trait associated with each sampled sequence was either the relevant production area (PA) of Atlantic salmon aquaculture in Norway (Ådlandsvik 2015) or a ‘sub-production area’. Production areas vary in size from 12 729 to 30 948 km^2^ and include between 67 and 258 farm sites. Sub-production areas were defined here as two subdivisions of each production area *i* (where *i* ranges from 1 to 13, defined in the order of production areas from the south to the north of the Norwegian coast), yielding units that are approximately half the size of the original production area. This was done by partitioning farms into two groups using a proximity based rule relative to neighbouring production areas: farms were assigned to sub-production area *i*-A or *i*-B depending on whether they were closer to the reference farm nearest the centroid of production area *i* − 1 or to the reference farm nearest the centroid of production area *i* + 1 (Fig. S1, with boundary rules applied for production areas 1 and 13).

We compiled a dataset of potential predictors thought to influence PMCV dispersal in Norway for 11 of the 13 production areas from which we had sequences, and the corresponding sub-production areas. Predictors included: (i) Euclidean distance between centroid points of each pair of (sub-)production areas (in kilometres); (ii) weighted density of wellboat connectivity networks between farms located in different (sub-)production areas, using a maximum delay of 1 day between a stop at a farm in one area and a subsequent stop at a farm in another area. Wellboats are specialized vessels used to transport live fish between farms. The weighted density of their connectivity network represents the intensity of wellboat movements between fish farms; (iii) weighted density of connectivity networks for other types of boats (excluding wellboats) between farms located in different (sub-)production areas, using a maximum delay of 1 day between a stop at a farm in one area and a subsequent stop at a farm in another area; (iv) density of on-land farms within each (sub-)production area, considering both the origin location of lineage spread and the destination; (v) density of at-sea farms within each area (both origin and destination); (vi) a binary production area similarity index, indicating whether the sequences came from adjacent production areas (1) or nonadjacent production areas (0); (vii) a binary sub-production area similarity index, indicating whether the sequences came from adjacent sub-production areas within the same production area (1) or different production areas or nonadjacent production areas (0); and (viii) a binary sub-production area similarity index, indicating whether the sequences came from adjacent sub-production areas belonging to different production areas (1) or from the same production area or nonadjacent sub-production areas (0). Details of data sources and further definitions for all predictors are summarized in Table S4.

We conducted multiple analyses to evaluate whether each covariate was associated with higher or lower PMCV lineage spread between locations. Each MCMC analysis comprised 250 million steps with sampling every 25 000 steps, using PMCV data exclusively from Norway. ‘Model A’ tested discrete traits representing production areas and included covariates (i), (ii), (iii), (iv), (v), and (vi). ‘Model B’ examined discrete traits representing sub-production areas and included covariates (i), (ii), (iii), (iv), (v), (vii), and (viii). Convergence of parameters was assessed visually using Tracer v1.7.1 (Rambaut et al. 2018), and results from two parallel MCMC runs were combined. Bayes factors (BFs) were computed to show the strength of evidence supporting each predictor’s inclusion in the GLM model (Kass and Raftery 1995, Suchard et al. 2001). BFs > 3 are typically considered suggestive, while BFs exceeding 20 indicate strong evidence supporting a predictor’s inclusion (Kass and Raftery 1995).

## 3. Results

### 3.1. Sequencing coverage

The proportion of the genome that could be successfully sequenced using our novel amplicon scheme varied based on the RT-qPCR Ct of PMCV. Almost all samples with Ct values below 26 achieved full coverage of the targeted region (94% of the full genome), while those with Ct values between 26 and 32 generally achieved high but variable coverage from 37% to 99% (mean coverage: 90%; median coverage: 98%). However, samples with Ct values above 32 generally failed to yield sufficient coverage (Fig. S2). We retained sequences with genome coverage exceeding 75%, resulting in a final dataset of 311 PMCV genomes, each 6590 bp in length: 222 from 75 fish farms in Norway and 89 sequences from 36 fish farms in Scotland. Cage information was available for 100 sequences. The number of PMCV sequences per farm ranged from 1 to 11 and per cage ranged from 1 to 5.

The number of sequences generated for each production area in Norway is listed in Fig. S1. We generated more sequences from southern Norway than northern Norway, consistent with a more extensive salmon farming industry in that area.

To confirm the accuracy of our nanopore-based approach, we sequenced a 460-nucleotide variable region (positions 2719–3178 bp) from nine PMCV samples using Sanger sequencing and observed no differences compared to the corresponding sequences obtained from our ONT data (Supplementary Data 1).

### 3.2. Root-to-tip regression analysis

Root-to-tip regression of the unrooted maximum likelihood tree of all PMCV genomes in this study revealed a relatively modest association between genetic distances and sampling dates (*R*^2^ = 0.11, correlation coefficient = 0.33) (Fig. S3A). When additional PMCV genomes from GenBank, the Faroe Islands, and Norway are included, the association becomes slightly stronger (*R*^2^ = 0.29, correlation coefficient = 0.54) (Fig. S3B). Both datasets showed sufficient temporal signal to support molecular clock analyses.

### 3.3. Phylodynamic analyses of piscine myocarditis virus

**Figure 1:**
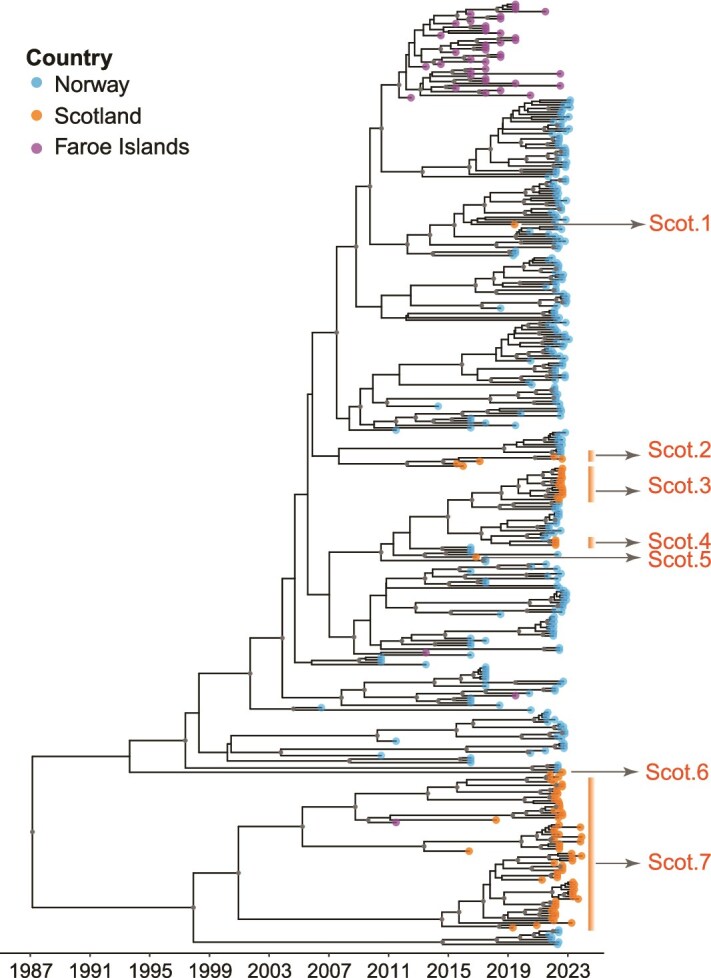
Time-scaled maximum clade credibility tree constructed using PMCV genome sequences generated in this study, together with PMCV whole genome sequences from the Faroe Islands and Norway obtained from GenBank (accession IDs listed in Tables S1 and S3). Tree tips are colour-coded by country of origin. ﻿Clades with posterior probabilities >80% are displayed with black dots. The arrow and text on the right side of the tree indicate the seven clades/singletons of Scottish PMCV, as summarized in Table 1.

The mean evolutionary rate estimated using BEAST was 3.8 × 10^−4^ substitutions per site per year (subs/site/year) (95% Highest Posterior Density (HPD) interval: 3.1 × 10^−4^–4.5 × 10^−4^). ORF3 demonstrates the highest substitution rate at 3.8 × 10^−4^ subs/site/year (95% HPD interval: 3.0 × 10^−4^–4.6 × 10^−4^), followed by ORF2 (RdRp) at 3.4 × 10^−4^ subs/site/year (95% HPD interval: 2.8 × 10^−4^ –4.0 × 10^−4^), and ORF1 (capsid) at 3.1 × 10^−4^ subs/site/year (95% HPD interval: 2.6 × 10^−4^–3.7 × 10^−4^). The estimated time of the most recent common ancestor (tMRCA) for all PMCV genomes from Scotland and Norway in our study, together with available sequences in GenBank from the Faroe Islands and Norway, was December 1986 (95% HPD: November 1975 to May 1996; Fig. 1). The estimated tMRCA for the clade comprising only Faroe Islands PMCVs is August 2012 (95% HPD: August 2011 to May 2013) (Fig. 1). The tMRCA for all Scottish and Norwegian PMCV sequences only in our study was estimated to be around May 1996 (95% HPD: June 1988 to December 2003; Fig. S4). Phylodynamic analysis of PMCV sequences from Norway and Scotland identifies two distinct clades. Clade I primarily consists of PMCV sequences (90%) from Norwegian fish farms (posterior support = 0.81), while Clade II mostly contains Scottish PMCV sequences (90%) (posterior support = 0.96). The estimated tMRCA for Clade I is July 2005 (95% HPD: January 2001 to May 2008) and for Clade II is April 2002 (95% HPD: April 1995 to September 2007) (Fig. S4).

We observed seven instances in which clades of Scottish samples, or Scottish singletons, were nested within Norwegian clades (Fig. 1). The 95% HPD interval for the closest ancestral node of PMCV from both Norway and Scotland and for the clades or singletons from Scotland are summarized in Table 1. The lower bound of the 95% HPD interval of the ancestral node and the upper bound of the 95% HPD interval for each Scottish clade (or the sampling date for a singleton) were used to infer the potential timing of PMCV introduction into Scotland. Scot.1 and Scot.5 are singletons, with inferred introduction time of 2014–9 and 2011–7, respectively. Clades Scot.2, Scot.3, and Scot.4 are estimated to have been introduced into Scotland during 2004–14, 2014–20, and 2016–21, respectively. Singleton Scot.6 and clade Scot.7 are less informative due to wide 95% HPD intervals, reflecting substantial uncertainty in their introduction times. These estimates indicate that PMCV was introduced into Scotland multiple times at different periods (Table 1).

**Table 1:** Estimated introduction time of PMCV lineages into Scotland, based on the mean estimated tMRCAs of relevant node pairs (clades or singletons from Scotland and their closest ancestral nodes). Seven clades/singletons are listed in the same top-to-bottom order as shown in Fig. 1. The 95% HPD interval for the closest ancestral node of PMCV from both Norway and Scotland, and for the clades or singletons from Scotland, is provided. The possible introduction time is defined as the interval between the lower 95% HPD boundary of the ancestral node and the upper 95% HPD boundary of the Scottish clade (or the sampling date for singletons), as highlighted in bold

**Clade ID**	**Number of tips in the clade**	**Mean and 95% HPD of the closest ancestral node**	**Mean and 95% HPD of the Scottish clade/sequence/date of sequence sampling (if singleton)**
Scot.1	1	Sep 2017; **Jan 2015**–Aug 2018	**Dec 2019**
Scot.2	5	Nov 2008; **Oct 2004**–Sep 2011	May 2013; Feb 2009–**Nov 2014**
Scot.3	14	Sep 2017; **Nov 2014**–Oct 2018	Sep 2019; Mar 2017–**Sep 2020**
Scot.4	3	Jan 2019; **Aug 2016**–Nov 2019	Apr 2020; Oct 2017–**Mar 2021**
Scot.5	1	Jul 2014; **Jan 2012**–Jun 2015	**May 2017**
Scot.6	1	Oct 1994; **Feb 1986**–Jul 2003	**Feb 2023**
Scot.7	64	Mar 1998; **Jul 1988**–Dec 2005	Apr 2001; Nov 1992–**Jun 2007**

**Figure 2:**
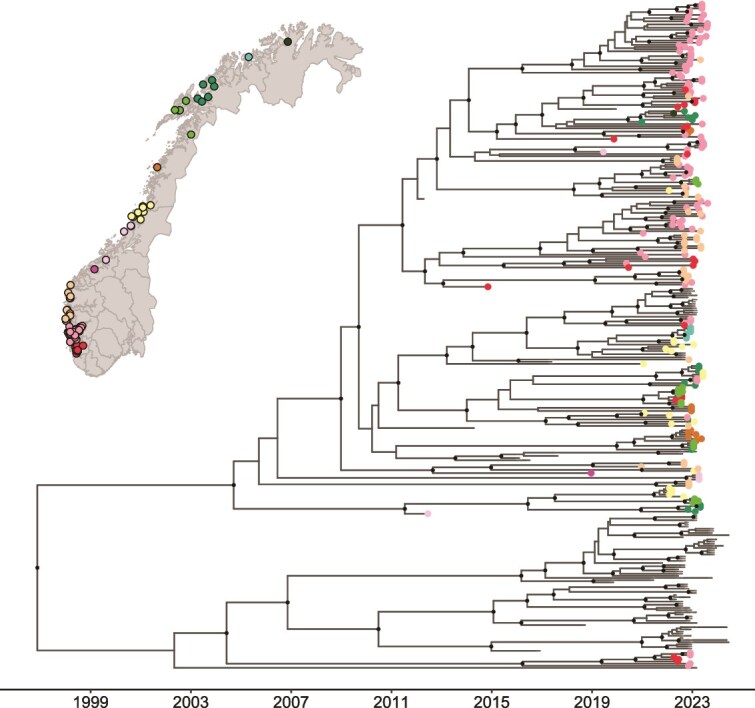
Time-scaled maximum clade credibility tree constructed from PMCV whole genome sequences in this study. Tree tips of Norwegian samples are colour-coded according to production areas, as indicated on the map. The map in the upper left shows sampling farm sites in Norway, with site locations anonymized by applying a random jitter of up to 0.1 degrees to both latitude and longitude. Clades with posterior probabilities > 80% are displayed with black dots.

**Figure 3:**
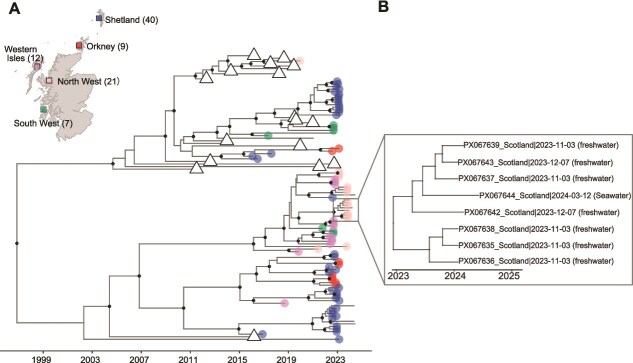
Time-scaled maximum clade credibility tree constructed from PMCV whole genome sequences in this study. (A) Tree tips for Scottish samples are categorized by production areas, as indicated on the upper-left map, while Norwegian samples are either clustered together (represented by triangles) or shown without tip markers. Numbers in parentheses indicate the number of sequences obtained from each production area. The shaded boxes on the map represent the relative locations of different production areas in Scotland. Clades with posterior probabilities > 80% are displayed with black dots. (B) Magnified view of the clade highlighted in (A).

PMCV sequences from the southern production areas of Norway tend to be more closely related to each other, and the same pattern is observed for the northern production areas (Fig. 2). The Scottish sequences in clade II are similarly grouped into geographically structured clades: PMCVs from Orkney (23%) and Shetland (73%) dominate one clade, while PMCV genotypes from the Western Isles (39%), Northern (46%), and Southern West Scotland (11%) are most common in the other clade (Fig. 3A). Interestingly, PMCV detected in salmon during the seawater phase was highly similar to PMCVs from a nearby freshwater broodstock site several months earlier, clustered in a clade (Fig. 3B). Both the freshwater and seawater sites are operated by the same company.

We used the Bayesian Tip-association Significance testing (BaTS) tool (Parker et al. 2008) to statistically quantify support for these groupings. Our analyses revealed significant phylogenetic clustering of PMCV sequences by country and production areas, as indicated by both Association Index (AI) and Parsimony Score (PS) statistics (*P* < .001) (Table 2). To mitigate potential biases from clustering of sequences obtained from the same farm, we tested the clustering of PMCV by production areas by randomly selecting only one genome per farm in Norway and obtained a similar result (Table S5).

**Table 2:** Results of the BaTS analysis assessing phylogeny–trait clustering by country, production area, and broodstock producer for PMCV samples from Norway and Scotland. The 5% and 95% intervals for both observed and null statistics are shown in brackets

**Country**	**AI**	**PS**
﻿Country (observed)	0.73 (0.55–0.98)	14.0 (11.0–17.0)
﻿Country (null mean)	11.46 (10.36–11.94)	97.55 (91.45–103.31)
﻿Country (*P*-value)	<.001^*^	<.001^*^
**Production area in Scotland** (all samples tested)	**AI**	**PS**
Production area (observed)	0.61 (0.53–0.69)	21.0 (18.0–24.0)
﻿Production area (null)	6.43 (5.55–7.31)	52.26 (48.74–55.60)
Production area (*P*-value)	<.001^*^	<.001^*^
**Production area in Norway** (all samples tested)	**AI**	**PS**
Production area (observed)	3.34 (3.27–4.24)	79.03 (74.0–84.0)
Production area (null)	19.75 (18.4–21.11)	163.78 (158.42–168.88)
Production area (*P*-value)	<.001^*^	<.001^*^
**Broodstock producer** (one sample per site tested)	**AI**	**PS**
Broodstock producer (observed)	3.75 (2.66–4.01)	32.93(30.0–36.0)
Broodstock producer (null)	4.04 (3.26–4.77)	34.86 (31.32–38.0)
Broodstock producer (*P*-value)	.26	.18

^*^ *P* < 0.001, indicating a statistically significant association based on 1,000 permutations.

At finer scales, we analysed the clustering and tMRCA of PMCV genomes from the same fish farm and from individual fish cages. In only about one-third of the fish farms (25/74), PMCV sequences from the same farm all clustered in a clade, with mean tMRCAs falling within 2 years of the sampling date (Table S6). More than 90% (23/25) of these farms had PMCVs collected on the same date. In contrast, for the remaining two-thirds (49 farms), the sequences were not clustered strongly by farm, and their tMRCAs ranged from 2 to 26 years before sampling. About half of these farms (24 farms) had PMCV sampled on the same day, while the other half had samples collected up to 8.2 years apart (Table S6).

At the cage level, PMCV sequences from 50% of the cages (15 out of 30) were highly similar, clustering together in clades and having estimated mean tMRCAs within 2 years of sampling. For these cages, samples were collected on the same day from 13 out of 15 cages. However, for the remaining 50% of the cages, PMCV sequences were more divergent, forming distinct clades on the tree, with tMRCAs ranging from over 2 to 26 years before sampling (Table S7). For these cages, samples were collected on the same day from 10 out of 15 cages. These patterns suggest that PMCV may have been introduced multiple times to the same farm.

**Figure 4:**
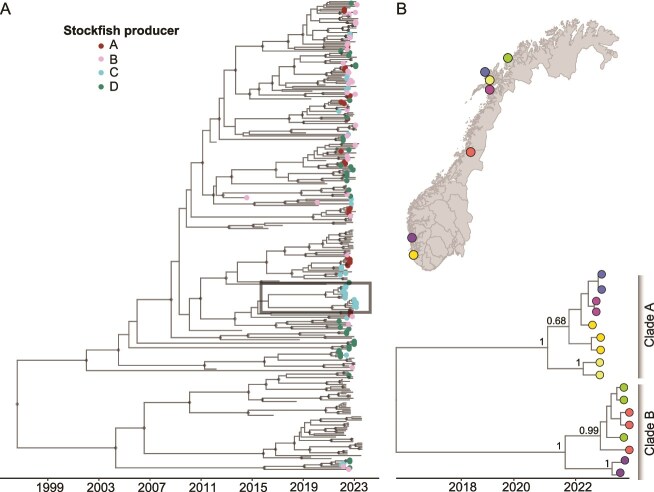
Time-scaled maximum clade credibility tree constructed from PMCV genome sequences in this study. (A) Tree tips are labeled by the anonymized broodstock producer associated with the fish samples, with unknown producers are shown without dots. (B) A magnified view of the clade of interest, highlighted by a grey rectangle. Tips in this section are categorized by sampling fish farm locations, as shown on the upper map. Locations on the map are anonymized by applying a jitter of up to 0.5 degrees to both latitude and longitude. Clades with posterior probabilities > 80% are displayed with black dots.

Most PMCV sequences do not cluster strongly based on the identity of the four producers who provided eggs to the sampled farms in Norway (Fig. 4A). This lack of significant association between PMCV clustering and the broodstock producer was also supported by the BaTS test (*P* > .1; Table 2). Due to limited access to broodstock producer information, we were unable to conduct a similar analysis for Scotland. Interestingly, we identified two well-supported clades (posterior support = 1), each comprising closely related PMCV sequences from farms located in geographically distant production areas. Clade A includes samples from four farms in production areas 2 and 9, while Clade B includes samples from farms in production areas 3, 7, and 10 (Fig. 4B). Except for two samples from production area 3 with no available broodstock producer information, all samples, whether in Clade A or Clade B, originated from farms that received eggs from the same broodstock producer (Fig. 4B). The tMRCA for Clade A is estimated to be July 2020 (95% HPD: April 2019 to June 2021), closely aligning with the producer-supplied egg production period from July to November 2020. The estimated tMRCA for Clade B is February 2021 (95% HPD: August 2019 to January 2022), which corresponds well with the egg production dates of those samples collected from production area 10 in September 2021. Egg production dates were not available for other samples in Clade B.

### 3.4. Spatial spread of piscine myocarditis virus

**Figure 5:**
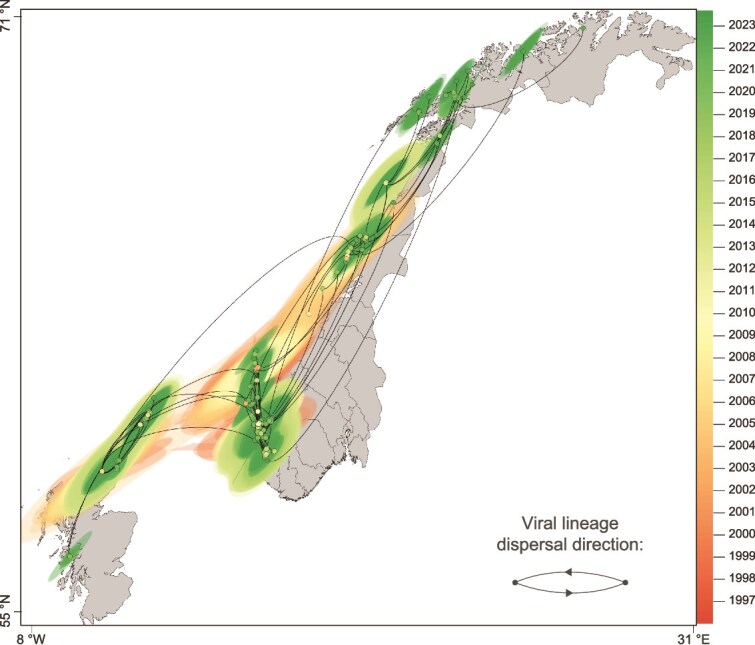
Spatiotemporal diffusion of PMCV lineages in Norway and Scotland. The maximum clade credibility tree is derived from continuous phylogeographic inference based on 1000 posterior trees. Nodes are colored according to their time of occurrence, with younger nodes plotted above old nodes. Shaded areas represent the 80% HPD interval and depict the uncertainty of the phylogeographic estimates for each node. Site locations on the map are anonymized with a random jitter of up to 0.1 degrees applied to both latitude and longitude.

We used a continuous phylogeographic model that reconstructs the dispersal history of PMCV lineages using the geographic coordinates of the sampled tips. Our continuous phylogeographic reconstruction suggests that the ancestral location of the PMCV lineages represented in our dataset is most consistent with southern Norway, followed by south to north dissemination within Norway (Fig. 5; Supplementary Data 2). Furthermore, the continuous phylogeographic analysis also revealed multiple introductions of PMCV from southern Norway into various regions of Scotland (Fig. 5). Our analysis indicated a weighted lineage dispersal velocity for PMCV lineages of ~24.45 km/year (95% HPD 22.58–27.89 km/year), calculated as the total great-circle distance travelled across all branches in the phylogeny, divided by the time elapsed on each branch (Table S8). The weighted diffusion coefficient was estimated to be 3364.64 km^2^/year (95% HPD 2990.69–3776.26 km^2^/year), reflecting the ‘diffusivity’ of PMCV across a 2D landscape (Table S8). This metric is known to be less sensitive to tip sampling density than the weighted lineage dispersal velocity.

### 3.5. Predictors for piscine myocarditis virus spread in Norway

**Figure 6:**
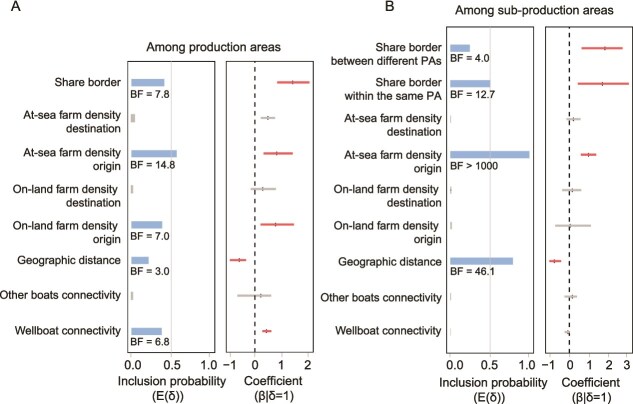
Predictors of PMCV spatial diffusion across production areas (A) and sub-production areas (B) in Norway. The inclusion probability E(δ) is the proportion of the posterior samples in which each predictor was included in the model (blue lines). The coefficient (β|δ=1) represents the contribution of each predictor on a log scale conditional to the migration rates of PMCV when the corresponding predictor is included in the model, with the 95% HPD interval of the log GLM coefficients (β) represented by horizontal lines (red) from the mean. Bayes factor (BF) support values > =3 are shown. Predictors without statistical support are shown in grey.

We employed a GLM extension of a discrete trait phylogeographic model to identify factors associated with PMCV dispersal between (sub-)production areas in Norway, summarizing our results in Fig. 6 and Table S9. Our first analyses in Model A focused on PMCV dispersal between production areas in Norway. The GLM results supported increased virus dispersal between production areas with a greater estimated connectivity by wellboats (BF = 6.8). Connectivity by other types of boats did not show a significant correlation with virus dispersal. PMCV dispersal was estimated to be higher between adjacent production areas than nonadjacent production areas (BF = 7.8) and negatively correlated with geographic distance (BF = 3.0). Additionally, PMCV dispersal was positively correlated with the density of at-sea farm sites at origin (BF = 14.8), indicating that production areas with higher at-sea farm densities were more likely to act as sources of viral spread. On-land farm density in the origin production areas also showed a positive correlation with viral dispersal, though we note that this is somewhat correlated with at-sea farm density (BF = 7).

We subsequently explored predictors of PMCV dispersal at smaller scales by dividing each production area into two similarly sized sub-production areas. In this analysis (Model B), the association between wellboat connectivity and virus dispersal was no longer significant (BF < 1). Instead, virus spread was greater with shorter geographic distances between sub-production areas (BF = 46.1) and between adjacent sub-production areas, regardless of whether they belonged to the same production area or different production areas. At-sea farm density at origin remained a significant positive predictor for virus dispersal between sub-production areas (BF > 1000). However, on-land farm density within sub-production areas was no longer supported as an important predictor (BF < 1).

### 3.6. Genetic diversity, recombination, and selection

Sequence identity within ORF1 ranges from 98% to 100% at the nucleotide level between pairs of sequences and from 98.5% to 100% at the amino acid level. For ORF2, nucleotide identity ranges from 97.8% to 100% and amino acid sequence identity from 98.9% to 100%. ORF3 exhibits the lowest sequence identity, with nucleotide identity ranging from 96.6% to 100% and amino acid identity from 94% to 100%. Recombination analysis conducted across the near full-length of all PMCV genomes did not detect any supported recombination events.

FEL and FUBAR tests were performed on all three ORFs to evaluate the presence of pervasive selection pressure on PMCV proteins. No positively selected sites were identified in ORF1 (capsid), with 118 sites under purifying selection. ORF2 (RdRp) exhibited a single positively selected site (AA position: 525), and 86 sites under purifying selection. ORF3 revealed three positively selected sites (AA position: 22, 122, and 222) and 34 sites under purifying selection out of 302 sites analysed. We also conducted a MEME test to examine episodic positive selection. ORF1 and ORF2 showed no sites subject to episodic positive selection, while ORF3 had two sites at AA positions 22 and 33 (Fig. S5; Table S10). The lack of correlation between amino acid sequence divergence and phylogenetic relationships suggests that positive selection at these sites does not appear to be associated with specific geographic regions (Fig. S6).

## 4. Discussion

### 4.1. Genetic diversity and evolutionary rate of piscine myocarditis virus

In this study, we substantially expand the availability of PMCV genomic sequences by generating 311 near-complete genomes, establishing one of the largest datasets of fish viral genomes to date. This dataset enabled a detailed investigation of PMCV evolution and genetic diversity and spread in farmed Atlantic salmon, and provided new insights relevant to disease surveillance and control in salmon aquaculture.

The mean evolutionary rate of PMCV was estimated to be 3.9 × 10^−4^ nucleotide subs/site/year, consistent with rates reported for dsRNA viruses (10^−3^–10^−4^ subs/site/year; Sanjuán 2012). Genetic diversity was low in both ORF1 and ORF2, in line with previous observations (Amono et al. 2025), and both genes showed strong purifying selection, indicating substantial functional constraint and suggesting that most amino acid changes are deleterious (Holmes 2009). While strong purifying selection is expected for ORF2, which encodes the conserved RNA-dependent RNA polymerase (de Farias et al. 2017), the similarly strong constraint observed in ORF1 is notable given that structural proteins can sometimes tolerate more variation.

In contrast, ORF3 and its encoded protein exhibited the highest nucleotide and amino acid diversity, the fastest estimated evolutionary rate, and the greatest number of codons under positive selection. Two positively selected amino acid sites (AA22 and AA222) have been reported previously, showing nonsynonymous mutations (Wiik-Nielsen et al. 2012, Tighe et al. 2019, Amono et al. 2025), while two additional sites (AA33 and AA122) identified here have not been described before. These novel substitutions may represent recently emerged variants in Norwegian and Scottish PMCV populations or mutations that were previously undetected due to limited sampling. These findings are consistent with emerging evidence that ORF3 may contribute to extracellular viral transmission by promoting host cell membrane interaction and cell lysis (Amono et al. 2025). Because sampling in our study is concentrated in 2022–3, our analyses are best interpreted as a high-resolution characterization of contemporary PMCV diversity and recent lineage dynamics. Inferences about deeper evolutionary history, including early diversification and longer-term selection regimes, are therefore less well resolved by the available temporal sampling and would be strengthened by the inclusion of whole genome sequences from earlier decades.

### 4.2. Spread of piscine myocarditis virus between countries

The estimated time of the most recent common ancestor (tMRCA) for PMCV genomes from Norway and Scotland in this study was 1996 (95% HPD: 1988–2003). This estimate does not fully encompass the first reported case of CMS in Norway in 1985 (Amin and Trasti 1988), likely due to the temporal skew in our dataset (~90% of genomes sampled in 2022–3). When older PMCV genomes from the Faroe Islands (2012–3) were included, the estimated tMRCA shifted back to approximately 1986 (95% HPD: 1975–96), underscoring the importance of historical sampling for robust molecular clock inference.

We identified multiple independent introductions of PMCV into Scottish production areas, occurring across different time periods. This is epidemiologically plausible given historical patterns of live animal movement and egg importation in salmon aquaculture. Scotland previously imported salmon ova, fry, parr, and smolts from Norway and Ireland and ova from Iceland (Marine Directorate 2025). Norway reportedly supplied a large portion of salmon eggs used in Scotland, but imports ceased in May 2019 following concerns about infectious salmon anaemia virus (ISAV), after which Iceland and Ireland became the primary egg suppliers (Marine Directorate 2025). Since 2016, Ireland has been the only exporter of fry, parr, and smolts to Scotland (Marine Directorate 2025). Phylogenetic clustering of Scottish sequences within Norwegian diversity is consistent with the possibility of introductions from Norway. However, because whole genome data from other salmon-producing regions remain sparse, we cannot definitively resolve transmission routes and unsampled sources may also have contributed. Supporting this uncertainty, a maximum likelihood phylogeny based on partial PMCV sequences revealed high similarity between Irish and western Scottish isolates (Fig. S7), suggesting potential virus exchange between Ireland and Scotland. Overall, while movement of eggs or live fish remains a plausible mechanism, establishing direct causality is challenging without more comprehensive genomic sampling and movement data across countries.

### 4.3. Vertical transmission of piscine myocarditis virus

The role of vertical transmission in PMCV epidemiology remains uncertain. PMCV RNA has been detected in progeny from infected broodstock, but viral loads are typically low and CMS symptoms are inconsistent (Wiik-Nielsen et al. 2012, Bang Jensen et al. 2019). Conversely, Mikalsen et al. (2020) reported no PMCV detection from first-feeding fry through the smolt stage. Notably, despite substantial egg exports from Norway to Chile and Iceland, PMCV has not, to date, been reported in either country (Mikalsen et al. 2020), although the extent to which this reflects true absence versus detection intensity is unclear.

In our Norwegian dataset, PMCV phylogeny showed no significant association with broodstock producer, and sequences sampled from the same farm or even the same cage frequently displayed distinct evolutionary histories, with inferred tMRCAs predating the hatching of the sampled fish. These findings suggest infected eggs are unlikely to represent the primary route of PMCV transmission. Nevertheless, we identified a well-supported clade in which genetically similar PMCV strains were detected across geographically distant farms receiving eggs from the same broodstock producers, with tMRCA estimates broadly consistent with egg production periods. This is compatible with egg-associated transmission, but alternative explanations, such as shared equipment, personnel, or other correlated management links, cannot be excluded. These results therefore support continued emphasis on egg disinfection and hatchery biosecurity while highlighting the need for targeted studies explicitly designed to test vertical transmission pathways.

### 4.4. Horizontal transmission of piscine myocarditis virus

Our analysis indicates that PMCV lineage movement is more extensive within Norway, Scotland, and the Faroe Islands than between them. This is consistent with previous genomic studies reporting strong clustering by country of origin and relatively limited inter-country mixing between Norway and the Faroe Islands (e.g. Amono et al. 2024). Within each region, we also detected clear phylogenetic structuring across production areas, supporting a geographic component to PMCV spread. Earlier studies in Norway and Ireland have reported weaker or less consistent spatial structuring (Wiik-Nielsen et al. 2013, Tighe et al. 2019, Amono et al. 2024), but the broader sampling and whole genome resolution here likely increase power to detect fine-scale patterns.

At farm and cage levels, PMCV genomes did not consistently form monophyletic clusters. This suggests repeated introductions and/or ongoing exposure to genetically distinct variants within sites, rather than a single introduction followed only by local onward spread, and is consistent with previously reported within-outbreak genomic heterogeneity of PMCV (Amono et al. 2024). Despite this within-site diversity, we found evidence of distance-dependent spread in Norway: farms in closer proximity were more likely to share related PMCV strains, and dispersal was consistently higher between adjacent areas than nonadjacent ones across spatial scales. These patterns may reflect waterborne transmission, movement of contaminated equipment and personnel between neighbouring farms, or involvement of biological vectors (Garseth et al. 2018). Waterborne transmission has been reported for other salmon viruses, including salmonid alphavirus (SAV) and infectious salmon anaemia virus (ISAV) (Viljugrein et al. 2009, Aldrin et al. 2010, 2011, Mardones et al. 2014), suggesting the plausibility of similar mechanisms for PMCV in salmon. In Scotland, the high genetic similarity between PMCV detected at a seawater farm and viruses detected at a nearby freshwater broodstock site several months earlier suggests potential viral exchange between freshwater and marine production environments. Further work integrating environmental sampling (including aquaculture wastewater) and hydrodynamic models would help quantify the contribution of water currents and waterborne transmission to PMCV spread.

### 4.5. Wild and escaped salmon, and other reservoirs

Wild fish species have been proposed as potential reservoirs or intermediate hosts for PMCV (Garseth et al. 2012). Although PMCV has also been detected at low prevalence in the wild salmon population (2 of 797 individuals), its potential role in viral maintenance and transmission cannot be excluded. Notably, PMCV sequences from wild salmon share >99% similarity with farmed salmon strains, indicating possible viral spread between wild and farmed populations (Garseth et al. 2012). Escaped farmed salmon may also contribute to viral circulation, as escape events occur at large scale in some years in Norway and Scotland (Føre and Thorvaldsen 2021, Marine Directorate 2025) and could facilitate transmission between wild and farmed populations (Madhun et al. 2015).

Efforts to identify alternative fish hosts for PMCV have largely yielded negative results, aside from PMCV detection in *Argentina silus* (Böckerman et al. 2011) and in two wrasse species used for sea-lice control (Scholz et al. 2018). Importantly, PMCV detected in wrasse was closely related to strains circulating in Atlantic salmon, supporting the possibility that wrasse could act as reservoirs or vectors, particularly if moved or reused between sites (Scholz et al. 2018). Because our sequencing focused on PMCV from farmed Atlantic salmon, expanding genomic sampling to include wild salmon, escaped salmon, cleaner fish, and other associated species (including invertebrates) will be important to better understand whether and how nonsalmon hosts contribute to PMCV persistence and spread.

### 4.6. Farm density and wellboat connectivity

Atlantic salmon are transferred to open-sea net pens following smoltification, and CMS is typically observed during the second year of the seawater phase (Garseth et al. 2018). Our findings show that areas with a higher density of at-sea farms are more likely to act as sources of PMCV spread to other regions. Continuous phylogeographic analysis further suggests a south-to-north spread of PMCV within Norway, consistent with the higher density of farms in southern production areas and higher reported CMS detection in earlier years (Moldal et al. 2025). Our dataset is enriched for PMCV genomes from southern Norway, which could partly reflect geographic sampling bias linked to diagnostic submission patterns. However, the concordance between farm density and inferred dispersal supports the interpretation that higher infection pressure in densely farmed southern regions contributes to PMCV spread.

Fish movements are a critical component of the salmon production cycle, and shipping activities have been identified as a major risk factor for disease spread (Murray et al. 2002). In our GLM analyses, wellboat connectivity was associated with increased PMCV dispersal between production areas, although the association was not supported at the sub-production area scale. This scale dependence suggests that wellboat movements may contribute more to long-distance dissemination, whereas local transmission may be dominated by short-range processes that require further investigation, such as waterborne spread or local operational contacts. Similar links between wellboat movements and viral spread have been reported for ISAV (Murray et al. 2002, Mardones et al. 2014) and incorporating high-risk wellboat movements can improve predictive modelling for SAV transmission (Haredasht et al. 2019). Given that PMCV is nonenveloped and may therefore be relatively environmentally stable, transmission *via* contaminated equipment or vessels is biologically plausible and warrants further investigation.

## 5. Conclusion

By generating one of the largest datasets of genomic sequences of a viral pathogen in aquaculture, we provide a comprehensive reconstruction of PMCV genetic diversity, evolutionary dynamics, and dispersal patterns in Norway and Scotland. Our analyses reveal significant spatial structuring of PMCV and highlight geographic proximity, at-sea farm density, and wellboat connectivity as important correlates of viral lineage spread, emphasizing that effective disease management must account for the spatial distribution, density, and connectivity of farms. In practical terms, our findings support risk based, multi-scale interventions, including enhanced surveillance and targeted sequencing in regions with high density of at-sea farms and in neighbouring areas following detection, coordinated area-level management such as synchronized stocking and fallowing where feasible to reduce infection pressure in spatially clustered farming regions, and strengthened wellboat biosecurity such as minimizing multi-site itineraries, optimizing route planning, and audited cleaning and disinfection between farms to reduce long distance dispersal. Given evidence consistent with repeated viral introductions at some sites, within-farm control should also prioritize cage-level compartmentalization and stricter management of equipment and personnel movements. More broadly, this study demonstrates how integrating whole genome sequencing with phylodynamic and phylogeographic approaches can inform surveillance design and targeted biosecurity to mitigate the spread of emerging pathogens in aquaculture systems.

## Supplementary Material

Supplementary_materials_veag020

## Data Availability

Sequences are available on NCBI GenBank under Accession numbers PX067395–PX067705. A phylogenetic tree with tip labels corresponding to Fig. 1 is provided in Supplementary Fig. S8. A video containing additional details related to Fig. 5 is provided as Supplementary Data 2. Metadata for each sequence are available in Supplementary Tables S1 and S3, with consistent naming across all datasets.
