# Supplementary material for: Molecular epidemiology and multi-scale drivers of piscine myocarditis virus dispersal in salmon aquaculture
Source: Virus Evol. 2026 Mar 28;12(1):veag020. doi: 10.1093/ve/veag020 (PMC13100901; doi:10.1093/ve/veag020)
Supplement: Supplementary_materials_veag020 [file supplementary_materials_veag020.zip › Supplementary materials/Figure S3A&B_MZ.pdf]

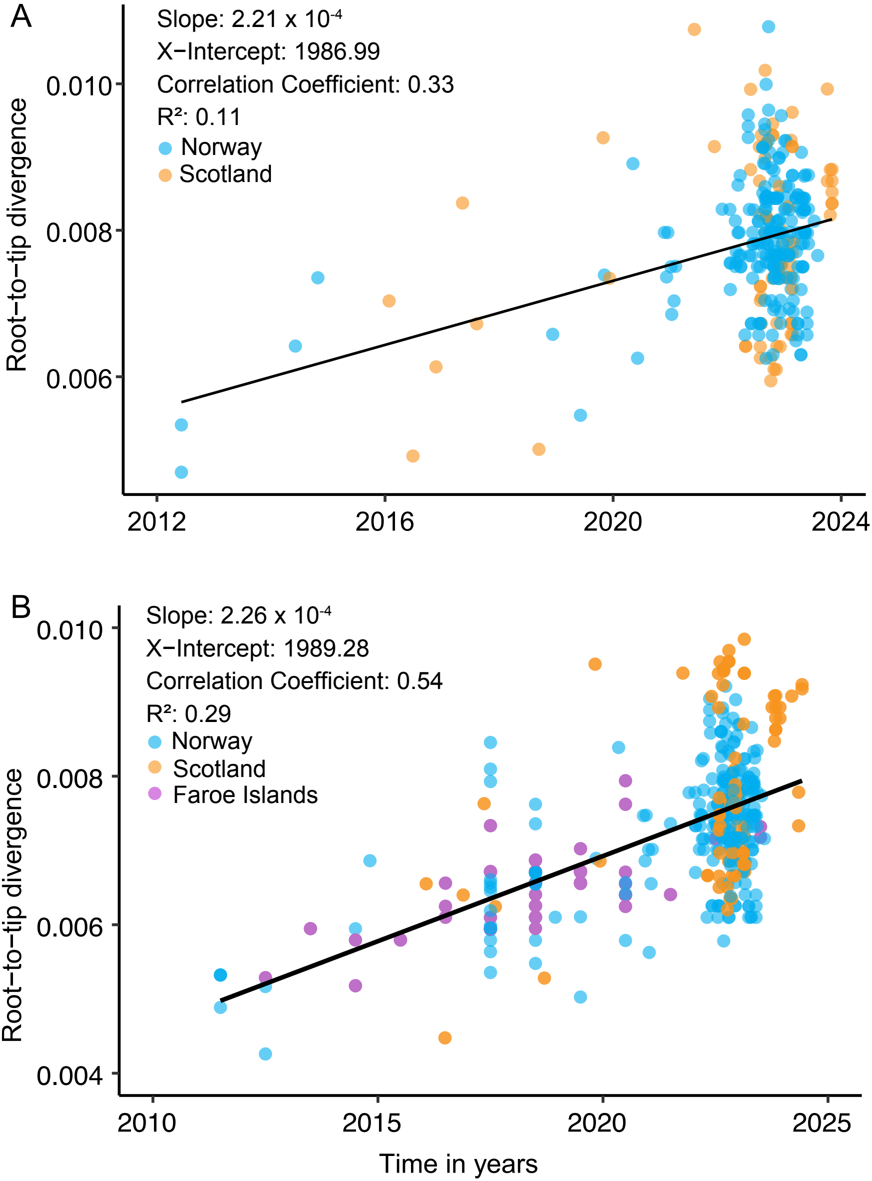

A
Slope: 2.21 x 10-4
X−Intercept: 1986.99
Correlation Coefficient: 0.33
R²: 0.11
Norway
Scotland
Root−to−tip divergence
0.010
0.008
0.006
2012
2016
2020
2024
B
Slope: 2.26 x 10-4
X−Intercept: 1989.28
Correlation Coefficient: 0.54
R²: 0.29
Norway
Scotland
Faroe Islands
Root−to−tip divergence
0.010
0.008
0.006
0.004
2010
2015
2020
2025
Time in years
