# Supplementary material for: Molecular epidemiology and multi-scale drivers of piscine myocarditis virus dispersal in salmon aquaculture
Source: Virus Evol. 2026 Mar 28;12(1):veag020. doi: 10.1093/ve/veag020 (PMC13100901; doi:10.1093/ve/veag020)
Supplement: Supplementary_materials_veag020 [file supplementary_materials_veag020.zip › Supplementary materials/Supplementary Figures_MZ.docx]

**Figure S1**. Map of Norwegian aquaculture production areas. Production areas are designated by Norwegian authorities, whereas the sub-production areas are defined here. Production area names and numbers are given in text, and polygons surround all active salmon farms in each production area based on public data shared via BarentsWatch (i.e., not only those farms that provide samples here). Red dots represent salmon farms in sub-production area A, while blue dots show farms in sub-production area B. To define sub-production areas, distances between every farm in production area *i* (where *i* ranges from 1 to 13, defined in the order of production areas from the south to the north of the Norwegian coast) and the centroids of production areas *i*+1 and *i*−1 were calculated. From these distances, the farm “a” closest to the centroid of production area *i*−1 and the farm “b” closest to the centroid of production area *i*+1 were identified. If *i* =1, farm ‘a’ was the farm furthest from the centroid of PA (*i* +1). Similarly, if *i* =13, ‘b’ was the farm furthest from the centroid of PA (*i* -1). Subsequently, the distances between all farms in production area *i* and these two reference farms were measured. Each farm was then assigned to sub-production area *i*-A if it was closer to the farm closest to production area *i*-1 than to the farm closest to production area *i*+1; otherwise, it was assigned to sub-production area *i*-B. Numbers in parentheses indicate the number of sequences obtained from each production area.

**Figure S2**. PMCV genome coverage obtained from samples with different RT-qPCR Ct values. Each red dot represents a PMCV genome, with the black line indicating a smoothed trend.

**Figure S3.** Root-to-tip regression of PMCV genome sequence dataset and associated statistics. A) PMCV sequences from this study. B) PMCV sequences from this study combined with sequences from the Faroe Islands obtained from NCBI datasets (accession IDs listed in **Table S3**).

**Figure S4**. Time-scaled maximum clade credibility tree constructed using PMCV genome sequences collected in Norway and Scotland between 2012 and 2023. Tree tips are color-coded by country of sampling. Clades with posterior probabilities greater than 80% are displayed with black dots. The mean root time of the most recent common ancestor (tMRCA) is shown, along with its 95% HPD interval. The shaded grey area represents the posterior probability densities for the 95% HPD interval of the estimated tMRCA.

**Figure S5**. Maximum likelihood estimates of synonymous (α) and non-synonymous substitution rates (β), calculated using Fixed Effects Likelihood (FEL) and Fast Unconstrained Bayesian Approximation (FUBAR), are shown for both negatively (green) and positively (red) selected amino acid sites across the three ORFs of PMCV. Fixed Effects Likelihood (FEL), Fast Unconstrained Bayesian Approximation (FUBAR). The line shows the estimates under the null model (α=β). Estimates above 10 are censored at this value to maintain clarity and prevent extreme values from distorting the scale.

**Figure S6**. PMCV phylogenetic tree displayed alongside amino acid diversity at five sites under positive selection detected by Fixed Effects Likelihood (FEL), Fast Unconstrained Bayesian Approximation (FUBAR) and MEME (Mixed Effects Model of Evolution). Tree tips are coloured by country, with the x-axis indicating time for the phylogenetic tree and amino acid sites are coloured by amino acid.

**Figure S7**. Maximum likelihood (ML) tree constructed from partial and concatenated PMCV sequences comprising 1353 bp from ORF1 (1478 bp-2235 bp) and ORF3 (5668 bp-6262 bp), generated in this study, along with PMCV sequences from GenBank for samples collected in the Faroe Islands, Norway and Ireland (accession IDs listed in **Table S3**). The tree was inferred using PhyML with 1000 ultrafast bootstrap replicates. Tree tips are colour-coded by country of origin (Faroe Islands, Norway and Ireland), and by production areas for Scottish PMCV. Major Scottish clades are annotated with their production area on the right side of the tree. Shaded regions highlight clusters of Irish PMCV sequences grouping with Scottish sequences from the Western Isles and south and north west coast of Scotland. Branch lengths are scaled in substitutions per site, and the tree is midpoint-rooted for visualization. Detailed information for each sequence is provided in Supplementary **Table S3**.

**Figure S8.** Time-scaled phylogenetic tree, the same as **Figure 1**, but with tree tip labels and posterior probability values shown on the tree.
