# Supplementary material for: Molecular epidemiology and multi-scale drivers of piscine myocarditis virus dispersal in salmon aquaculture
Source: Virus Evol. 2026 Mar 28;12(1):veag020. doi: 10.1093/ve/veag020 (PMC13100901; doi:10.1093/ve/veag020)
Supplement: Supplementary_materials_veag020 [file supplementary_materials_veag020.zip › Supplementary materials/Supplementary Tables_MZ.docx]

**Table S1. ﻿**GenBank accession numbers and metadata for PMCV sequences generated in this study.

**Table S2.** Multiplex PCR primers designed to generate amplicons of PMCV.

**Table S3. ﻿**Accession numbers of PMCV sequences from NCBI GenBank.

**Table S4.** Description of predictors included in the discrete trait phylogeography with generalised linear model.

| **Data** | **Source** | **Summary** |
| --- | --- | --- |
| i: Geographic distance | BarentsWatch | Euclidean distance between centroid points of pairs of production areas/sub-production areas, as shown by the polygons in Figure S1 (in kilometres). The coordinates (latitude and longitude) of a centroid point for a given production area correspond to the average latitude and the average longitude of all farms within that production area. |
| ﻿ii: Wellboat connectivity | BarentsWatch | The ratio of the number of wellboat visits between pairs of farms and the number of possible edges, where an edge represents a directed pair of farms that could be visited by the same wellboat on a given day during 2022 and 2023. This ratio reflects the intensity of actual wellboat traffic relative to the total number of potential farm-to-farm connections. |
| iii: Other boats connectivity | BarentsWatch | The ratio between the number of other boats (except wellboat) visits between pairs of farms and the number of possible edges, where an edge represents a directed pair of farms that could be visited by the same boat on a given day during 2022 and 2023. This ratio reflects the intensity of actual other boats traffic relative to the total number of potential farm-to-farm connections. |
| iv: On-land farm density (origin & destination) | BarentsWatch | Number of on-land (freshwater phase) farms within a production area/sub-production area divided by the size of the area, as shown by the polygons in **Figure S1**. |
| v: At-sea farm density (origin & destination) | BarentsWatch | Number of at-sea farms within a production area/sub-production area divided by the size of the area as shown by the polygons, as shown in **Figure S1**. |
| vi: Share border  (production areas) | BarentsWatch | A binary production area similarity index, indicating whether the sequences came from adjacent production areas (1) or non-adjacent production areas (0). |
| vii: Share border (sub-production areas) within the same production area | BarentsWatch | A binary production area similarity index, indicating whether the sequences came from adjacent sub-production areas within the same production area (1) or adjacent sub-production areas in different production areas or non-adjacent production areas (0). |
| viii: Share border (sub-production areas) between different production areas | BarentsWatch | A binary production area similarity index, indicating whether the sequences came from adjacent sub-production areas belong to different production areas (1), or adjacent sub-production areas within the same production areas or non-adjacent sub-production areas (0). |

**Table S5.** Results of the BaTS analysis assessing the phylogeny-trait clustering by production area. Tests are conducted with a subset dataset of only one genome per farm site. The 5% and 95% intervals for the observed and null statistics are included in brackets. AI: association index; PS: parsimony score.

| **Production area in Scotland**  (one sample per site tested) | **AI** | **PS** |
| --- | --- | --- |
| Production area (observed) | 1.27 (0.82 – 1.64) | 16.77 (15.0 – 19.0) |
| Production area (null) | 2.91 (2.28 – 3.46) | 23.28 (20.88 – 25.24) |
| Production area (*P* value) | < 0.001* | < 0.001* |

| **Production area in Norway**  (one sample per site tested) | **AI** | **PS** |
| --- | --- | --- |
| Production area (observed) | 2.40 (2.0 – 2.82) | 27.77 (25.0 – 30.0) |
| Production area (null) | 4.43 (3.75 – 5.06) | 36.33 (33.70 – 38.50) |
| Production area (*P* value) | < 0.001* | < 0.001* |

**Table S6. The mean estimated tMRCAs of PMCV from the same fish farm.**

**Table S7. The mean estimated tMRCAs of PMCV from the same fish cage.**

**Table S8.** PMCV dispersal statistics estimated from continuous phylogeographic inference using sequences from both Norway and Scotland.

| **Statistics** | **﻿Median value** | **﻿95% HPD** |
| --- | --- | --- |
| ﻿Mean branch dispersal velocity | 47.33 km/year | 31.38, 85.92 |
| Weighted branch dispersal velocity | 24.45 km/year | 22.58, 27.89 |
| Mean diffusion coefficient | 5992.94 km^2^/year | 3558.94, 16658.46 |
| Weighted diffusion coefficient | 3364.64 km^2^/year | 2990.69, 3776.26 |

**Table S9. ﻿**Bayes factor results for the phylogeographic generalised linear models.

| **Factors** | **﻿Model A** | **﻿Model B** |
| --- | --- | --- |
| ﻿Wellboat connectivity | 6.8 | 0 |
| Other boats connectivity | 0.2 | 0.1 |
| Geographic distance | 3.0 | 46.1 |
| On-land farm density origin | 7.0 | 0.2 |
| ﻿On-land farm density destination | 0.2 | 0.2 |
| At-sea farm density origin | 14.8 | 2099.0 |
| At-sea farm density destination | 0.4 | 0.1 |
| Share border | 7.8 | NA |
| Share border within the same production area (PA) | NA | 12.7 |
| Share border between different production areas (PAs) | NA | 4.0 |

**Table S10.** Amino acid sequences at positively selected sites in ORF2 and ORF3 of PMCV.
