# Supplementary material for: Molecular epidemiology and multi-scale drivers of piscine myocarditis virus dispersal in salmon aquaculture
Source: Virus Evol. 2026 Mar 28;12(1):veag020. doi: 10.1093/ve/veag020 (PMC13100901; doi:10.1093/ve/veag020)
Supplement: Supplementary_materials_veag020 [file supplementary_materials_veag020.zip › Supplementary materials/Figure S1_MZ.pdf]

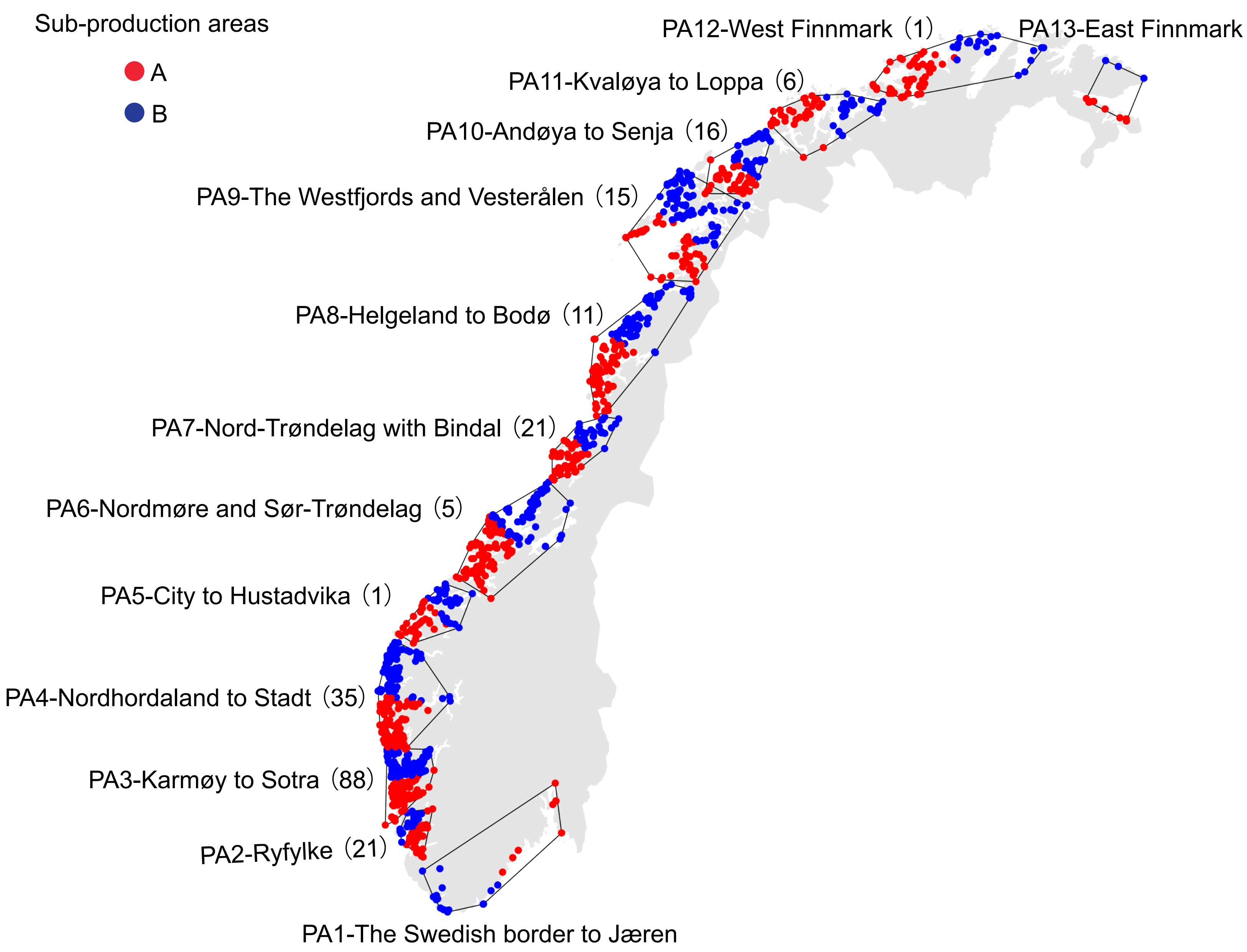

Sub-production areas
A
B
PA12-West Finnmark（1）
PA13-East Finnmark
PA11-Kvaløya to Loppa（6）
PA10-Andøya to Senja（16）
PA9-The Westfjords and Vesterålen（15）
PA8-Helgeland to Bodø（11）
PA7-Nord-Trøndelag with Bindal（21）
PA6-Nordmøre and Sør-Trøndelag（5）
PA5-City to Hustadvika（1）
PA4-Nordhordaland to Stadt（35）
PA3-Karmøy to Sotra（88）
PA2-Ryfylke（21）
PA1-The Swedish border to Jæren
