# Supplementary figures and images for: Molecular epidemiology and multi-scale drivers of piscine myocarditis virus dispersal in salmon aquaculture
Source: Virus Evol. 2026 Mar 28;12(1):veag020. doi: 10.1093/ve/veag020 (PMC13100901; doi:10.1093/ve/veag020)

## Country

- Norway
- Scotland

**Clade I**

**Clade II**

1995 1999 2003 2007 2011 2015 2019 2023

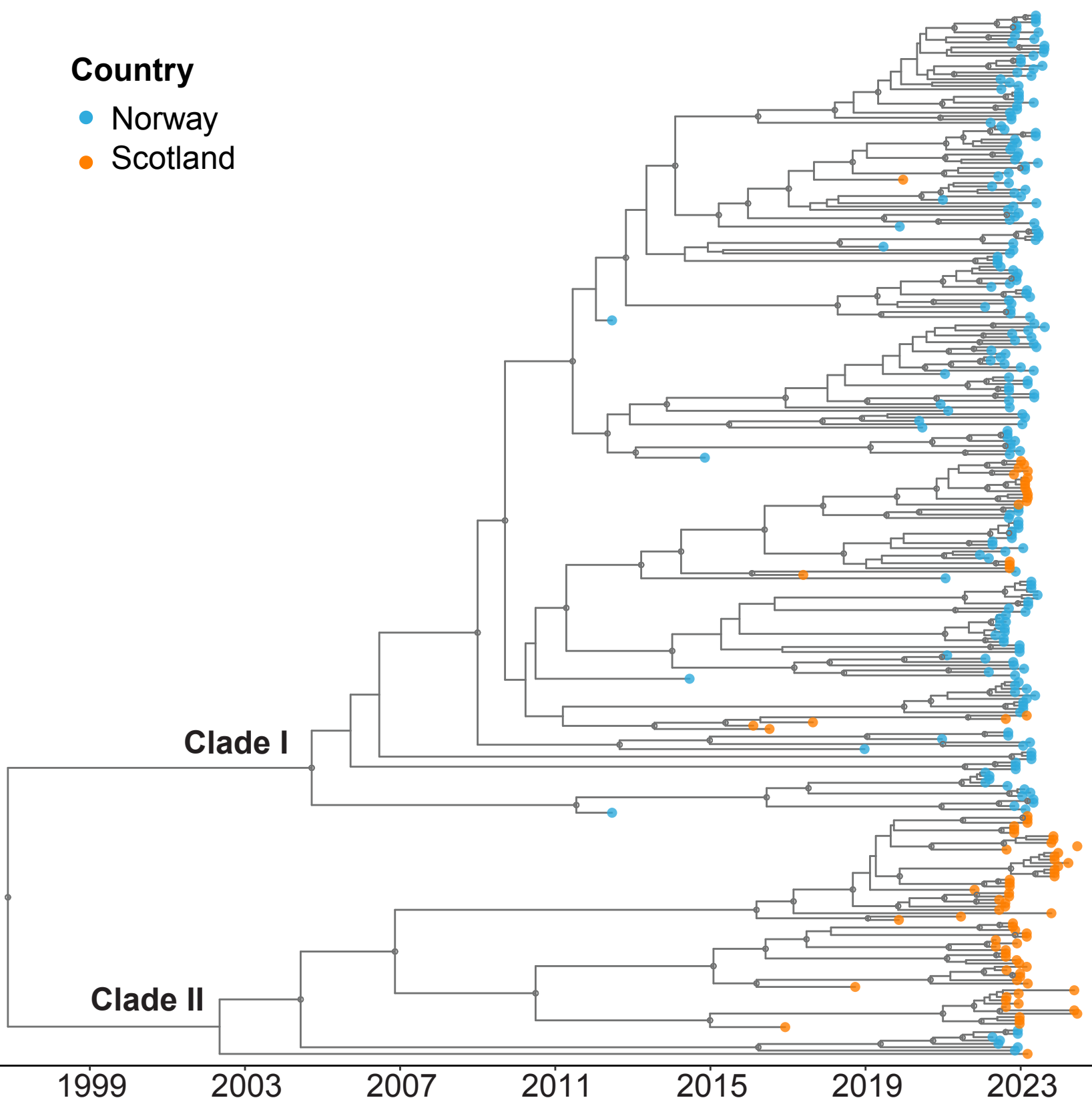

Supplement: Supplementary_materials_veag020 [file supplementary_materials_veag020.zip › Supplementary materials/Figure S4_MZ.pdf]

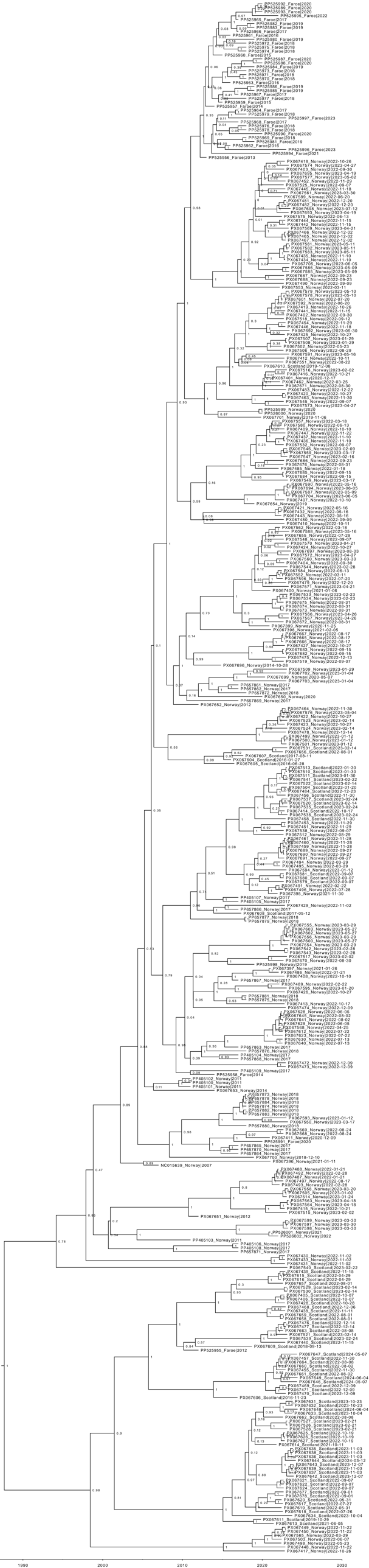

Supplement: Supplementary_materials_veag020 [file supplementary_materials_veag020.zip › Supplementary materials/Figure S8_MZ.pdf]

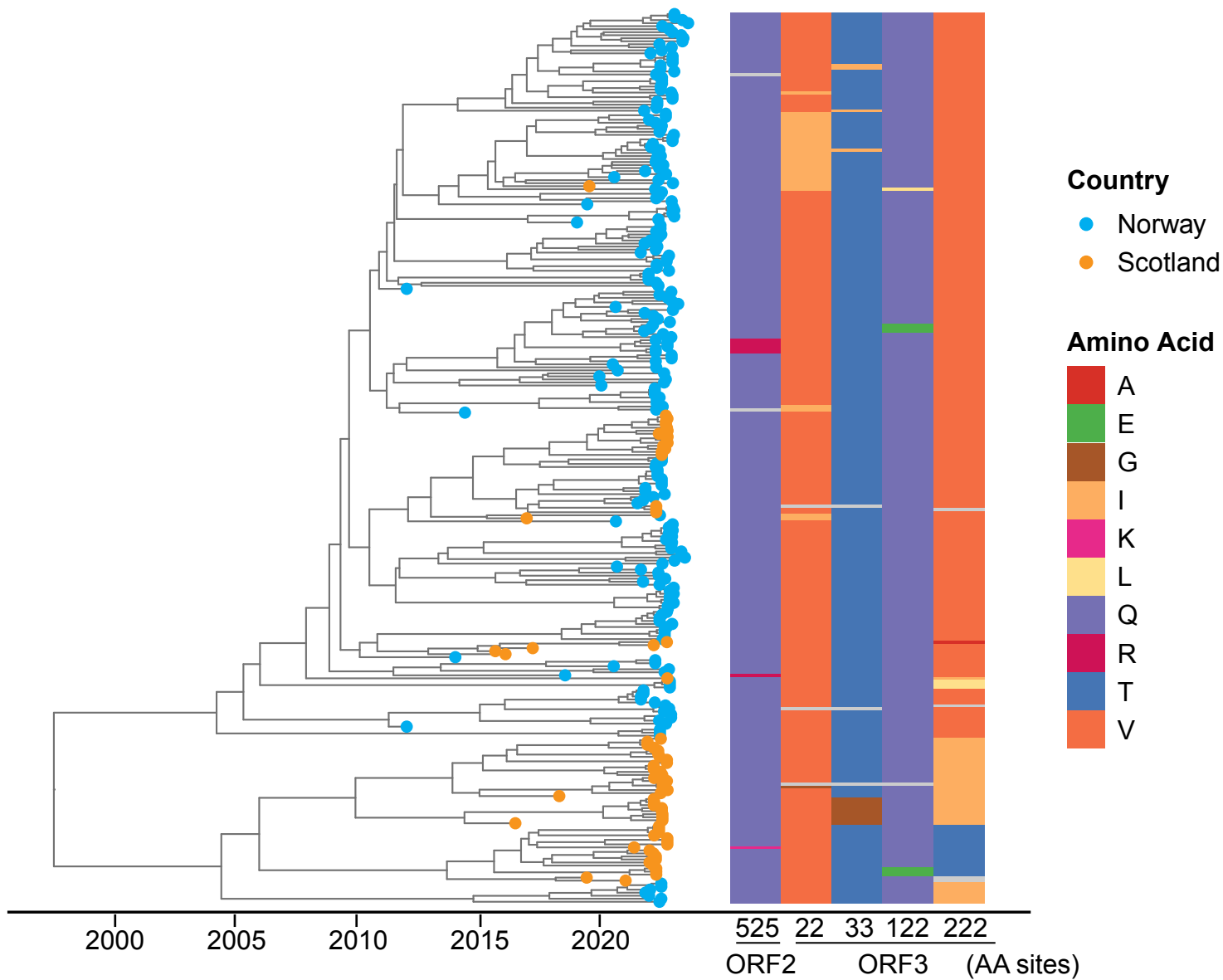

Supplement: Supplementary_materials_veag020 [file supplementary_materials_veag020.zip › Supplementary materials/Figure S6_MZ.pdf]

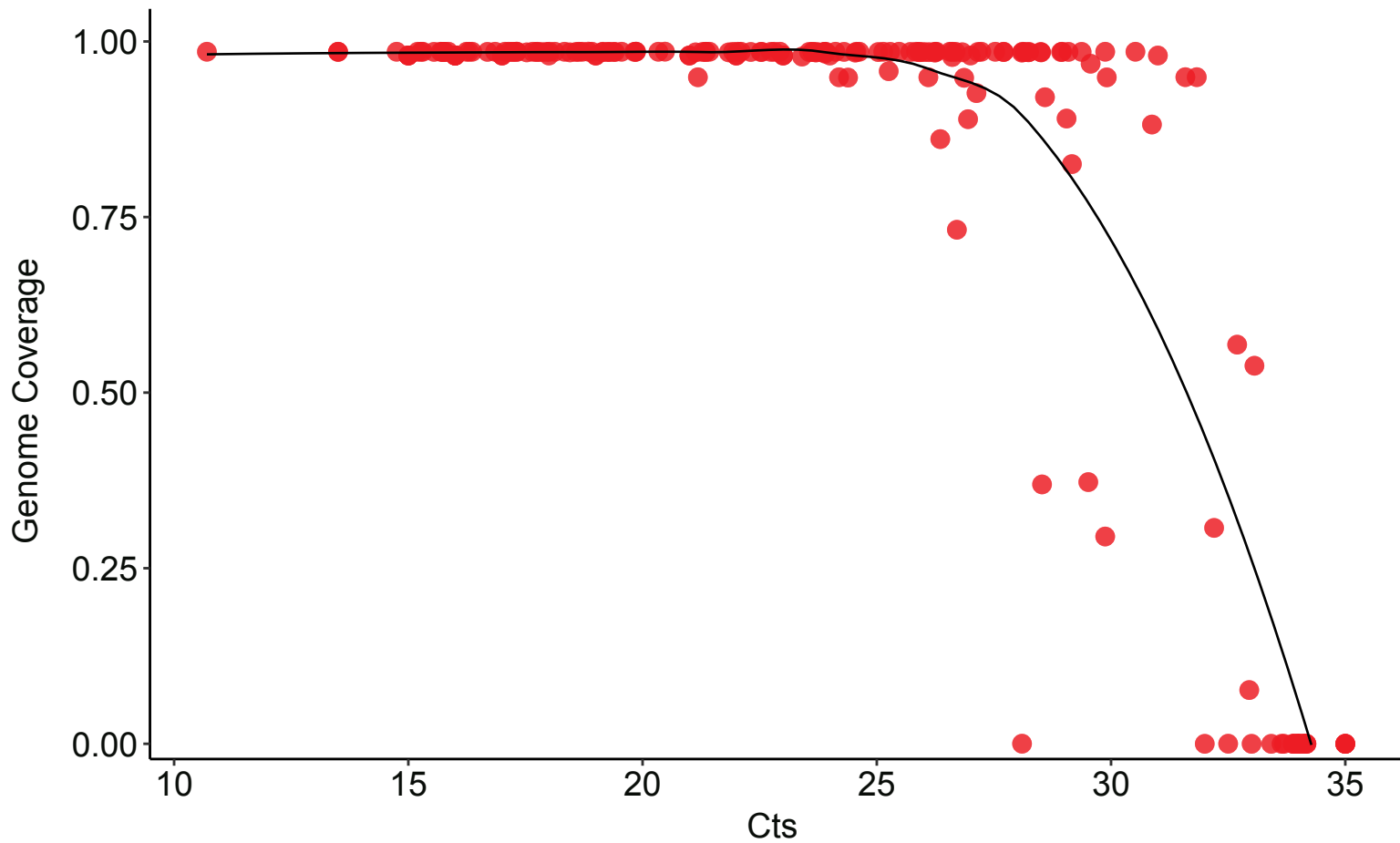

Supplement: Supplementary_materials_veag020 [file supplementary_materials_veag020.zip › Supplementary materials/Figure S2_MZ.pdf]

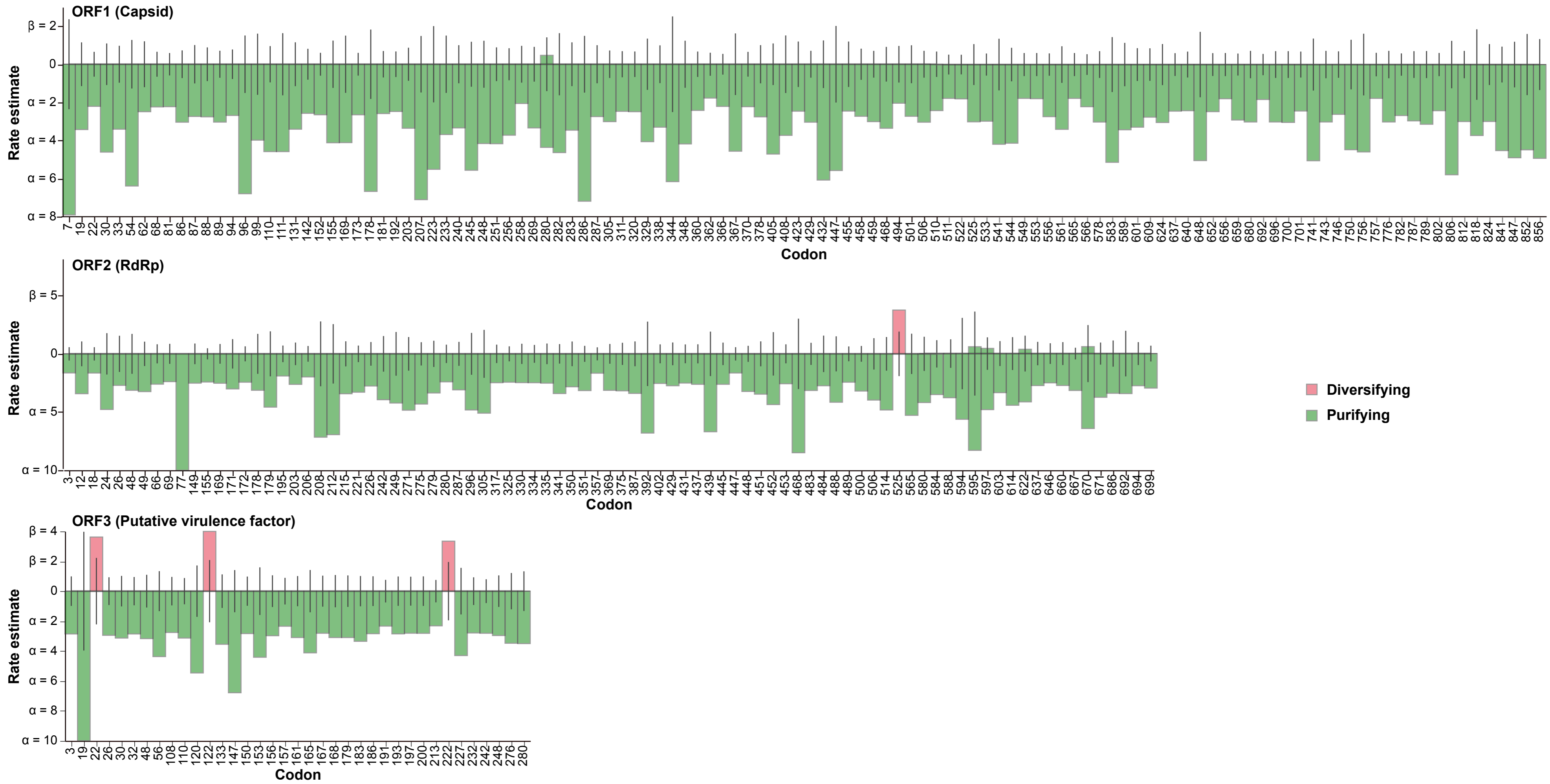

Supplement: Supplementary_materials_veag020 [file supplementary_materials_veag020.zip › Supplementary materials/Figure S5_MZ.pdf]

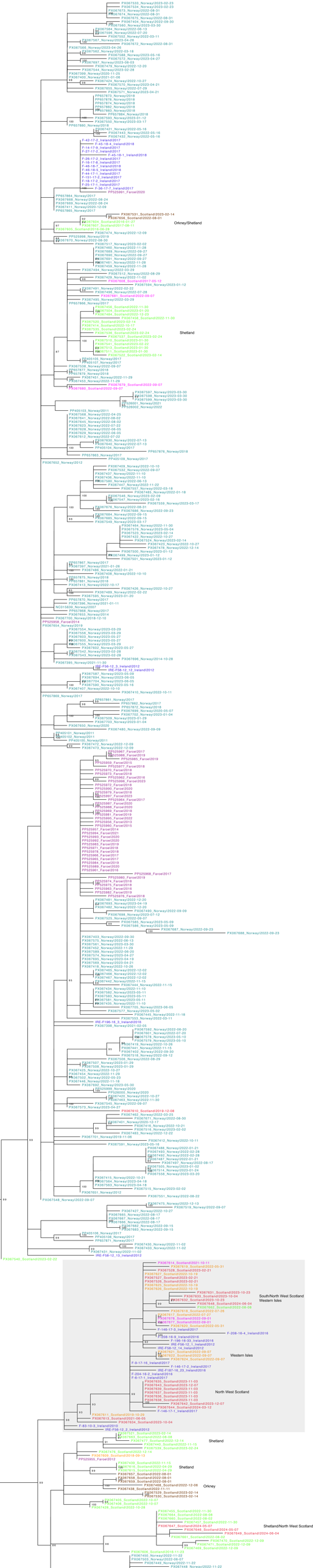

Supplement: Supplementary_materials_veag020 [file supplementary_materials_veag020.zip › Supplementary materials/Figure S7_MZ.pdf]
